# Effects of loxoprofen on the apical root resorption during orthodontic tooth movement in rats

**DOI:** 10.1371/journal.pone.0194453

**Published:** 2018-04-25

**Authors:** Taeko Yamamoto, Masato Kaku, Hiromi Sumi, Yuka Yashima, Jin Izumino, Kotaro Tanimoto

**Affiliations:** Department of Orthodontics and Craniofacial Developmental Biology, Hiroshima University Graduate School of Biomedical Sciences, 1-2-3 Kasumi, Minami-ku, Hiroshima, Japan; Charles P. Darby Children's Research Institute, 173 Ashley Avenue, Charleston, SC 29425, USA, UNITED STATES

## Abstract

Studies have revealed that severe apical root resorption during tooth movement is caused by the noninfective inflammatory reaction of apical root tissues. We hypothesized that loxoprofen can suppress apical root resorption during tooth movement. Cyclic tensile force (CTF) of 10 kPa was applied to the human pulp cells for 48 hours by the Flexcell Strain Unit. Loxoprofen (10 and 100 μM) was added to the culture cells, and expression of cyclooxygenase (COX)-1, COX-2, interleukin (IL)-1β, receptor activator of nuclear factor kappa-B ligand (RANKL), tumor necrosis factor (TNF)-α, and macrophage colony-stimulating factor (M-CSF) were examined. To determine the effects of loxoprofen sodium on apical root reabsorption during tooth movement, the upper first molars of 7-week-old rats were subjected to mesial movement by 10*g* force for 30 days with or without the oral administration of loxoprofen. Gene expression and protein concentration of COX-1, COX-2, IL-1β, TNF-α, RANKL and M-CSF were significantly higher in the CTF group than in the control group. However, these levels were decreased by loxoprofen administration. After orthodontic tooth movement, the expression of IL-1β, TNF-α, RANKL and M-CSF decreased in the loxoprofen group than in the control group by immunohistochemical staining. In comparison to control group, less number of odontoclasts and a decrease in the amount of apical root resorption was observed in the loxoprofen group. Many osteoclasts became visible on the pressure side of the alveolar bone in the both groups, and the amount of tooth movement did not show a significant difference. These findings demonstrate that severe apical root resorption may be suppressed by loxoprofen administration, without a disturbance of tooth movement.

## Introduction

Orthodontic treatment is useful to correct malocclusion; however, root resorption occurs occasionally. Severe root resorption is difficult to predict and repair. Root resorption caused by this mode of treatment has been established to be related to many factors, such as orthodontic force [1], systemic conditions [2], treatment period [3], malocclusion type [4,5], and patient age [2]. Root resorption is also associated with inflammatory reactions of pulp tissue at the root apex because orthodontic force is recognized as trauma [6,7]. It was reported that human pulpal blood decreased when continuous tipping forces were applied [7], and continuous heavy orthodontic forces can make susceptible to apical pulp tissue to pulp necrosis via the rupture of blood vessels [6]. So, it is suspected that apical pulp tissues may receive orthodontic force such as tensile strain through the apical foramen during orthodontic tooth movement. Moreover, it has been reported that root resorption is more frequently observed in vital teeth than in pulpectomized teeth [8,9]. Recently, we proved that tensile force can enhance the expression of interleukin (IL)-1β, receptor activator of nuclear factor kappa-B ligand (RANKL), tumor necrosis factor (TNF)-α, and macrophage colony-stimulating factor (M-CSF) in human pulp cells [10]. The expression of these factors at the time of investigational tooth movement was high in the apical pulp tissue of rats; however, they were not detected in the pulp without tooth movement. There was a significantly higher number of odontoclasts around the root apices in vital teeth than in pulpectomized teeth [11]. Furthermore, the quantity of internal root reabsorption was significantly higher in vital teeth than in pulpectomized teeth [10, 11]. Therefore, we concluded that noninfective inflammatory reaction of the apical root pulp tissue may contribute to apical root resorption during orthodontic tooth movement. Based on these observations, we hypothesized that apical root resorption will be inhibited by blocking cyclooxygenase (COX) activity using non-steroidal anti-inflammatory drugs (NSAIDs). Therefore, in the present investigation, we probed the consequence of loxoprofen on the expression of COX-1, COX-2, IL-1β, TNF-α, RANKL, and M-CSF in the human dental pulp cells after the application of cyclic tensile force (CTF). Furthermore, we investigated the effect of loxoprofen administration on apical root resorption during orthodontic tooth movement.

## Materials and methods

### Cell cultures

All teeth were obtained from orthodontic patients who provided gave consent. Ethical approval for this study was obtained from the Ethics Committee of Hiroshima University. The explanation of this study was informed and written informed consent was obtained from participants. Protrusion method was used for cultivating human dental pulp cells in accordance with a previous study [12]. The medium used was alpha minimum essential medium, supplemented with 250 μg/mL of amphotericin B (Nacalai Tesque, Kyoto, Japan), 32 U/mL of penicillin-G (Meiji Seika, Tokyo, Japan), and 60 μg/mL of kanamycin (Meiji Seika, Tokyo, Japan) at a temperature of 37°C and humidity of of 5% CO_2_. Culturing medium was changed twice a week. The subculturing was done by treating the cells with 0.25% trypsin/ethylenediaminetetraacetic acid (EDTA) followed by platting at 3 × 10^5^ cells per 100-mm culture dish. Under all experimental conditions the cells used were all between the fourth and sixth passages.

### Cyclic tensile force application

**Fig 1:**
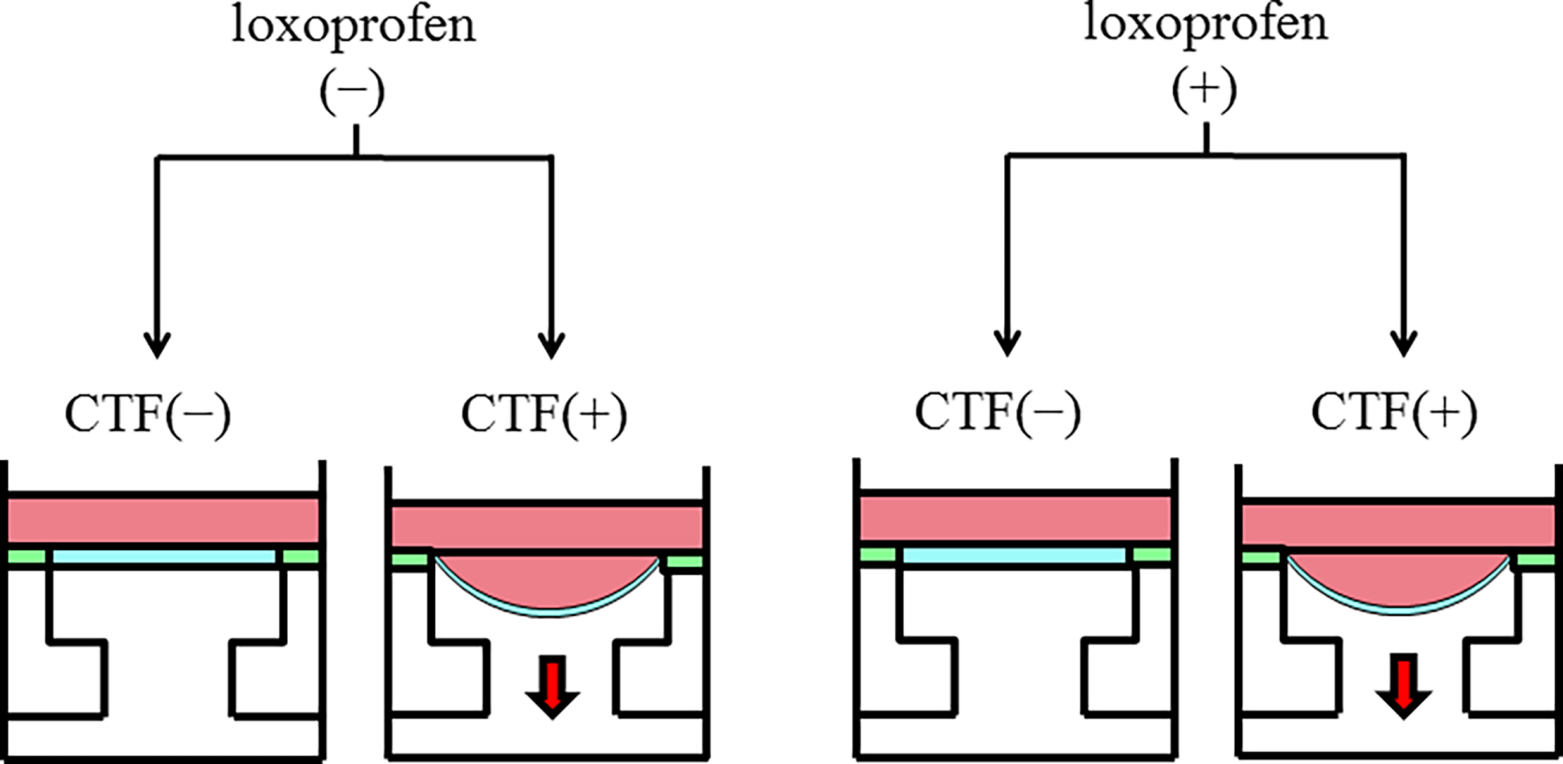
CTF application and addition of loxoprofen sodium. A maximal cell lengthening of 20% is created by a stain unit (FX-2000) with or without loxoprofen sodium.

A stain unit (FX-2000, Flexcell International Co, Hillsborough, NC, USA) that comprised of a vacuum unit and a valve controlled by a computer program was used in this study (Fig 1). Pulp cells (1 × 10^5^) cultivated on an adjustable membrane bottom were put through CTF created by a computer-administered implementation of sinusoidal negative pressure. This resulted in a maximal cell lengthening of 20% at the edge of the wells, along with declination toward the center. Subsequently the cells were incubated in a humidified incubator under an atmosphere of 5% CO_2_ at 37°C. To examine the expression of COX-1, COX-2, IL-1β, TNF-α, RANKL and M-CSF messenger mRNAs, and the protein concentrations of IL-1β, TNF-α, RANKL and M-CSF, a tensile force of 10 kPa was loaded at a frequency of 30 cycles per minute for 48 hours (CTF group) according to our previous study [10]. The cells without application of CTF were used as control.

### Addition of loxoprofen sodium

Cells were incubated with loxoprofen sodium (10 or 100 μM; Medisa Shinyaku Inc., Osaka, Japan) and were prepared to assess the effects of CTF of 10 kPa on the amounts of COX-1, COX-2, IL-1β, TNF-α, RANKL and M-CSF for 48 hours. We also prepared the non-CTF group with 100-μM loxoprofen sodium (Fig 1).

### RNA extraction and complementary DNA generation

QuickPrep Total RNA Extraction Kit (Amersham Biosciences, Tokyo, Japan) was used in the total RNA extraction. 1 μg of total RNA was used in the cDNA synthesis using oligo(dT)20 primer (Toyobo) and a ReverTra Ace-α First-strand cDNA Synthesis Kit (Toyobo).

### Primers

The following primers were used.

COX-1; (SIGMA-ALDRICH, ID:6775083) 5’-ATAATCGGGGGTTTGGCAA-3’ (forward), 5’-CAGATGCGAGCAGGAGTAGG-3’(reverse),

COX-2; (SIGMA-ALDRICH, ID:6775079) 5’-TCCTAGTCCTCATCGCCCTC-3’ (forward), 5’-AGATTAGTCCGCCGTAGTCG-3’(reverse),

IL-1β [13]; 5’-CTCAGGTGTCCTCGAAGAAATCAA-3’ (forward), 5’-GCTTTTTTGCTGTGAGTCCCG-3’(reverse),

TNF-α [14]; 5’-CCCCAGGGCTCCAGGCGGTGCTTGT-3’(forward), 5’-GGAGACGGCGATGCGGCTGATGGTG-3’(reverse);

RANKL [15];5’-TCAGAAGATGGCACTCACTG-3’(forward), 5’-AACATCTCCCACTGGCTGTA-3’(reverse);

M-SCF [16]; 5’-GGCCATGAGAGGCAGTCCGAGGG-3’(forward), 5’-CACTGGCAGTTCCACCTGTCTGTC-3’(reverse).

Glyceraldehyde-3-phosphate dehydrogenase (G3PDH) primer (Rever Tra Ace-α, First-strand cDNA Synthesis Kit, Toyobo) was used as a control primer; 5’-TGAAGGTCGGAGTCAACGGATTTGGT-3’ (forward), 5’-CATGTGGGCCATGAGGTCCACCAC-3’ (reverse).

### qPCR analysis

Quantitative real-time polymerase chain reaction (PCR) was carried out using SYBR Green I assay in conjunction with an ABI Prism 7700 sequence detection system (Biosystems, Foster City, CA, USA). A template cDNA of volume 1 μL was used during PCR under the following parameters: an initial denaturation step at 94°C for 30 seconds, followed by an annealing step at 62°C for 30 seconds, and a final step of primer extraction at 72°C for 30 seconds for 33 cycles. Each reaction was replicated three times.

The PCR results were analyzed using a cycle threshold (Ct) value, which helps in recognizing a cycle based on the fluorescence signal received. The relative expression levels of the COX-1, COX-2, IL-1β, TNF-α, RANKL, and M-CSF signals were normalized and shown relative to the G3PDH signals.

### Measurement of IL-1β, TNF-α, RANKL, and M-CSF protein concentrations

Conditioned medium from cultured dental pulp cells, with or without CTF implementation, was collected and cleared at 2000 rpm for 5 minutes. The protein concentration of IL-1β and TNF-α (Quantikine Human IL-1β Immunoassay Kit, Quantikine Human TNF-α Immunoassay Kit, R&D Systems, Inc, Wien, Austria), RANKL (BI-20452 AmplisRANKL Human Immunoassay Kit, BiomedicaGuppe, Inc), M-CSF (Quantikine Human M-CSF Immunoassay Kit, R&D Systems, Inc, Minneapolis, MN, USA) was measured using the quantitative sandwich enzyme immunoassay technique following the guidelines from the manufacturer. The usual process was followed to generate the standard curves, and the experiment was performed in triplicate.

### Experimental animals and treatment

**Fig 2:**
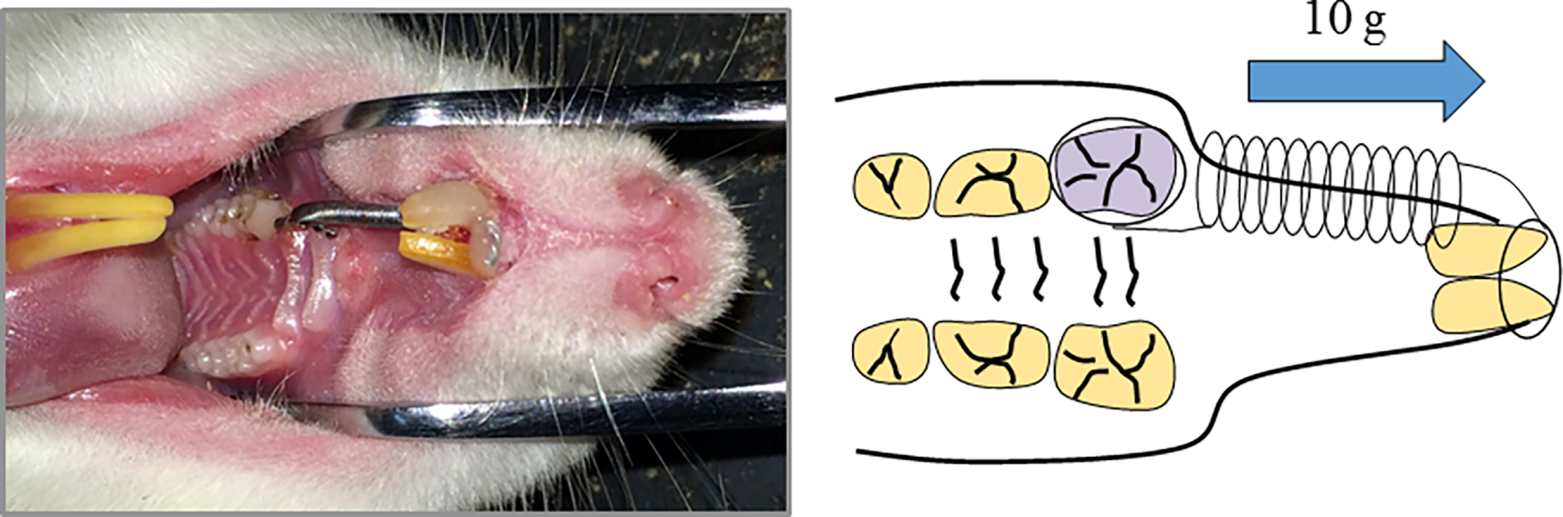
Experimental tooth movement and administration of loxoprofen. The upper right molar were moved mesially by closed-coil spring with magnitude at 10 g for 30 days.

Ten 7-week-old Wistar rats were used in this experiment. All animal procedures were approved by the Ethics Committee of the Hiroshima University Faculty of Dentistry (A-15-116). The animals were handled in compliance to the ethical mandate for experiments involving animals listed by the Ethics Committee of the Hiroshima University Faculty of Dentistry and they were alleviated suffering by medetomidine (1.0 mg/ml, Kyoritsu Seiyaku Corporation, Tokyo, Japan), midazolam (5.0 mg/ml, SANDOZ, Tokyo, Japan) and butorphanol (5.0 mg/ml, Meiji Seika Pharma, Tokyo, Japan). An investigational device with a closed-coil spring was boned onto the upper right molar and incisor. The molar was subjected to mesial movement for 30 days with magnitude at 10g (Fig 2).

**Fig 3:**
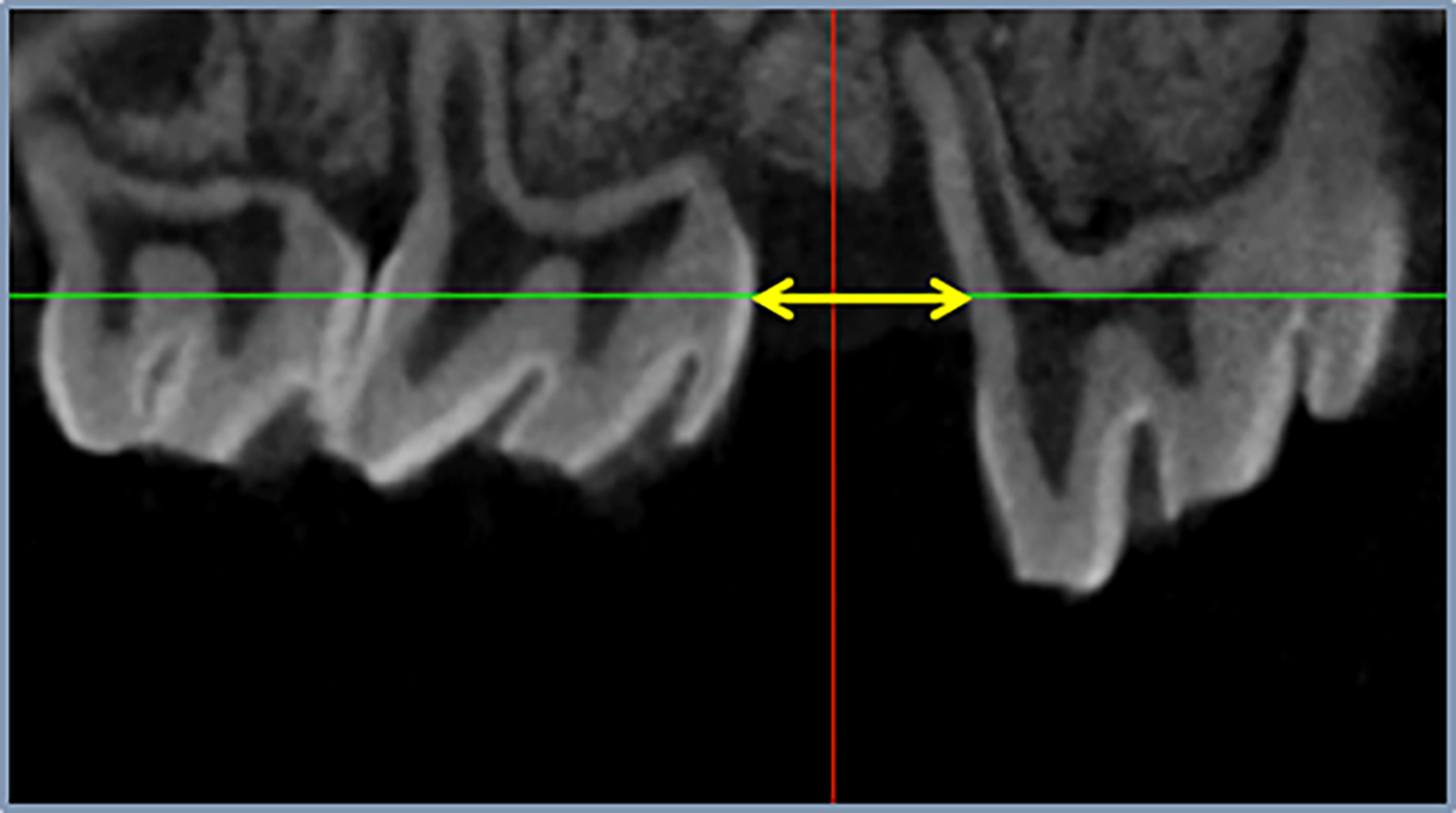
Observation of tooth movement. After 30 days, the amount of tooth movement was measured by means of computed tomography.

The animals were randomly split into two groups of five rats each (control group and loxoprofen group). The control group underwent orthodontic treatment for 30 days without any pharmacological treatment. The experimental group underwent oral administration of loxoprofen (1 mg/kg/day) by a feeding needle (loxoprofen group). Non- tooth movement without pharmacological treatment group is used as negative control for immunohistochemistry. After 30 days, the distance between the upper first and second molar was measured for calculating the amount of tooth movement by computed tomography (Sky Scan 1176, Bruker micro CT, Kontich, Belgium, Fig 3) and data viewer (Bruker micro CT, Kontich, Belgium).

Specimens were fixed in 4% paraformaldehyde, decalcified in 14% EDTA (pH 7.4) for 30 days, and implanted in paraffin. Premaxillary bones, including the upper molars, were excised into frontal sections of 5-μm thickness. For immunohistochemical staining, the sections were deparaffinized and endogenous peroxidase activity was quenched by incubation in 3% H_2_O_2_ in methanol for 30 minutes at room temperature. After washing in phosphate buffered saline, the sections were incubated with polyclonal rabbit-anti-human IL-1β (abcam; working dilution, 1:50), polyclonal goat-anti-human TNF-α (R&D systems, Inc.; working dilution: 1:100), polyclonal mouse-anti-human RANKL (abcam; working dilution, 1:100), and polyclonal goat-anti-human M-CSF (Santa Cruz Biotechnology; working dilution: 1:100) overnight at 4°C. IL-1β was stained using Histofine Simple Stain MAX-Po (Rabbit) kits (Nichirei, Tokyo, Japan), TNF-α and M-CSF were stained using Histofine Simple Stain MAX-Po (Goat) kits (Nichirei, Tokyo, Japan) and RANKL was stained using Histofine Simple Stain MAX-Po (Mouse) kits (Nichirei, Tokyo, Japan) according to the manufacturer's protocol. The sections were rinsed with PBS and final color reactions were performed using the substrate reagent 3, 3′-diaminobenzidine tetra-hydrochloride and aminoethylcarbazole and then counter-stained with hematoxylin.

**Fig 4:**
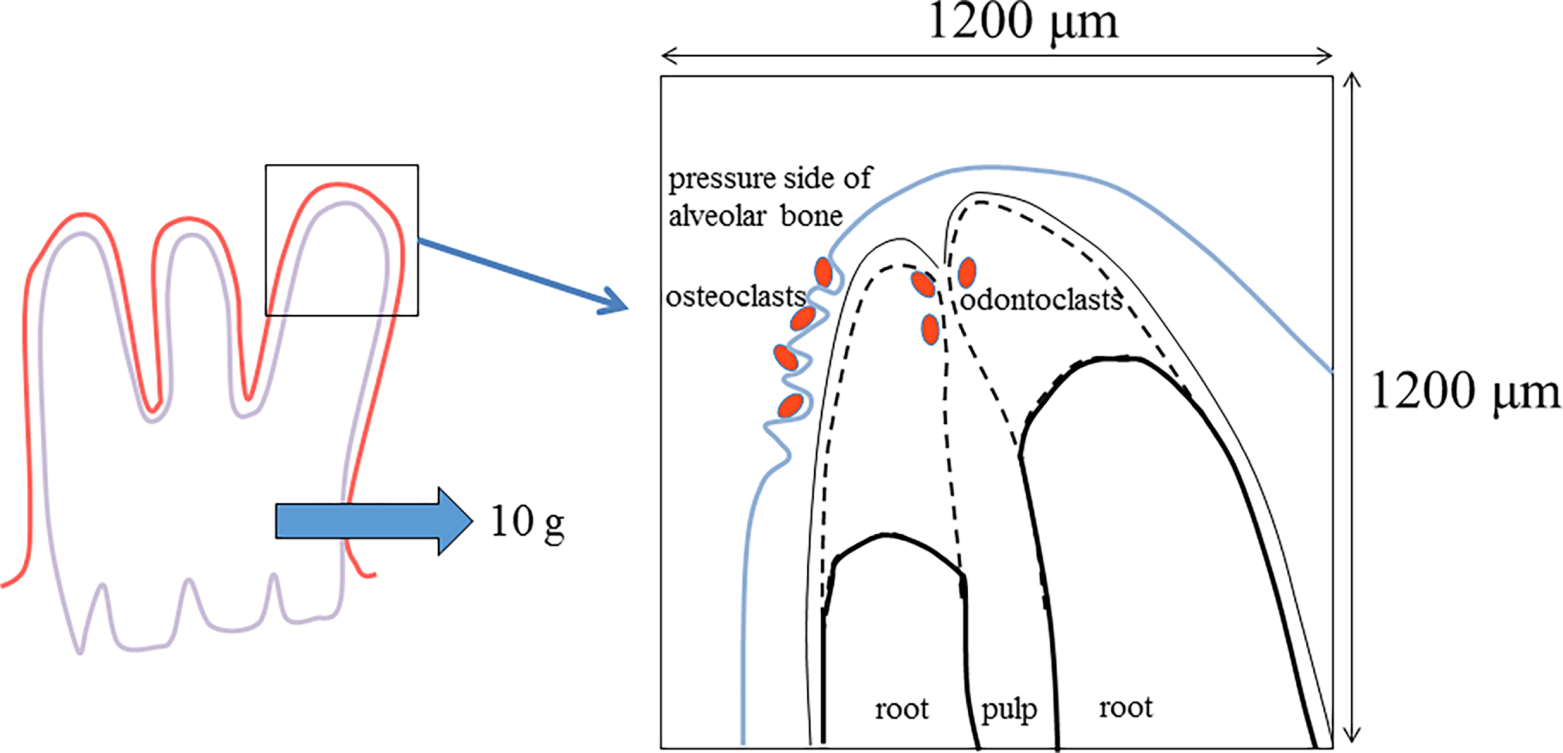
Observation of bone and root resorption area. The number of osteoclasts, odontoclasts and the amount of root resorption were examined at square area (1200 × 1200 μm^2^) in root apex.

The histological sections were stained with tartrate-resistant acid phosphatase (TRAP) and counterstained with hematoxylin. TRAP-positive multinucleated osteoclasts on the pressure side of alveolar bone and odontoclasts that appeared at the root apex (measuring range: 1200 × 1200 μm^2^, Fig 4) were counted on five sections at 35-μm intervals for each specimen according to previous studies [10, 11].

For evaluating the amount of root resorption at the apical area (measuring range: 1200 × 1200 μm^2^, Fig 4), five sections from each sample were used. Digitized photomicrographs were obtained using software on a computer (NIH Image, Bethesda, MD, USA).

### Statistical treatment

Statistical significance of the mRNA expression level and protein concentration was assessed by analysis of variance followed by the fisher’s methodology. We used Student’s *t*-test to evaluate statistical differences in the amount of tooth movement, number of osteoclasts and odontoclasts, and area of root resorption between the control and loxoprofen groups. p < 0.05 was adjudged statistically significant.

## Results

### Effects of loxoprofen on the expression of COX-1, COX-2, IL-1β, TNF-α, RANKL, and M-CSF mRNAs after application of CTF

**Fig 5:**
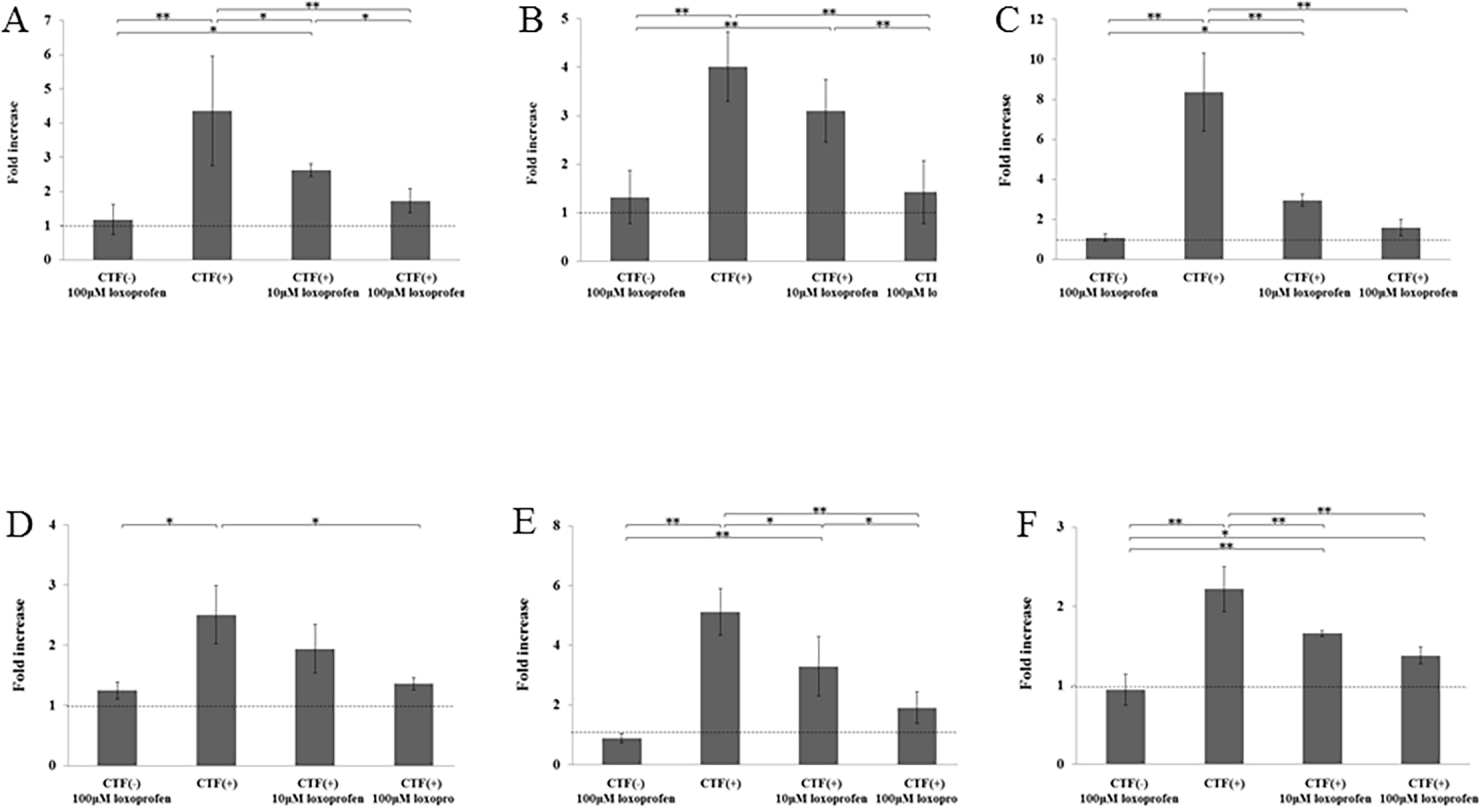
The effects of loxoprofen sodium on the expression of COX-1, COX-2, IL-1β, TNF-α, RANKL and M-CSF mRNA in pulp cells stimulated by a 10-kPa tensile force for 48 hours. (A) Dotted line indicates the expression of COX-1, (B) COX-2, (C) IL-1β, (D) TNF-α, (E) RANKL, and (F) M-CSF mRNAs in the control group. COX-1, IL-1β, RANKL, and M-CSF mRNA levels were decreased significantly in the 10- and 100-μM loxoprofen groups. COX-2, TNF-α mRNA level was decreased significantly in the 100-μM loxoprofen group compared with the CTF group (*P< 0.05, **P< 0.01, N = 4).

The gene expression level of all factors was significantly increased by the application of CTF. The gene expression level of COX-1 mRNA in the 10- and 100-μM loxoprofen groups decreased by 1.7- and 5.2-fold, respectively, compared with the CTF group (Fig 5A) and the expression of COX-2 mRNA in the 10- and 100-μM loxoprofen groups decreased by 1.3- and 2.8-fold, respectively, compared with the CTF group (Fig 5B). The gene expression of IL-1β mRNA in the 10- and 100-μM loxoprofen groups decreased by 2.8- and 5.3-fold, respectively, compared with the CTF group (Fig 5C). The expression of TNF-α mRNA decreased by 1.3- and 1.8-fold in the 10- and 100-μM loxoprofen groups in comparison to the CTF group (Fig 5D). The gene expression levels of RANKL in the 10- and 100-μM loxoprofen groups were decreased approximately by 1.5- and 2.7-fold, respectively, compared with the CTF group (Fig 5E). The expression of M-CSF in the 10- and 100-μM loxoprofen groups were decreased by 1.3- and 1.6-fold, respectively, in comparison to the control group (Fig 5F). The reduction in gene expression levels of these factors loxoprofen was dose-dependent. There was no significant difference between the control group and CTF(-) with 100-μM loxoprofen group in the expression of all these factors (Fig 5A–5F).

### Protein expression

**Fig 6:**
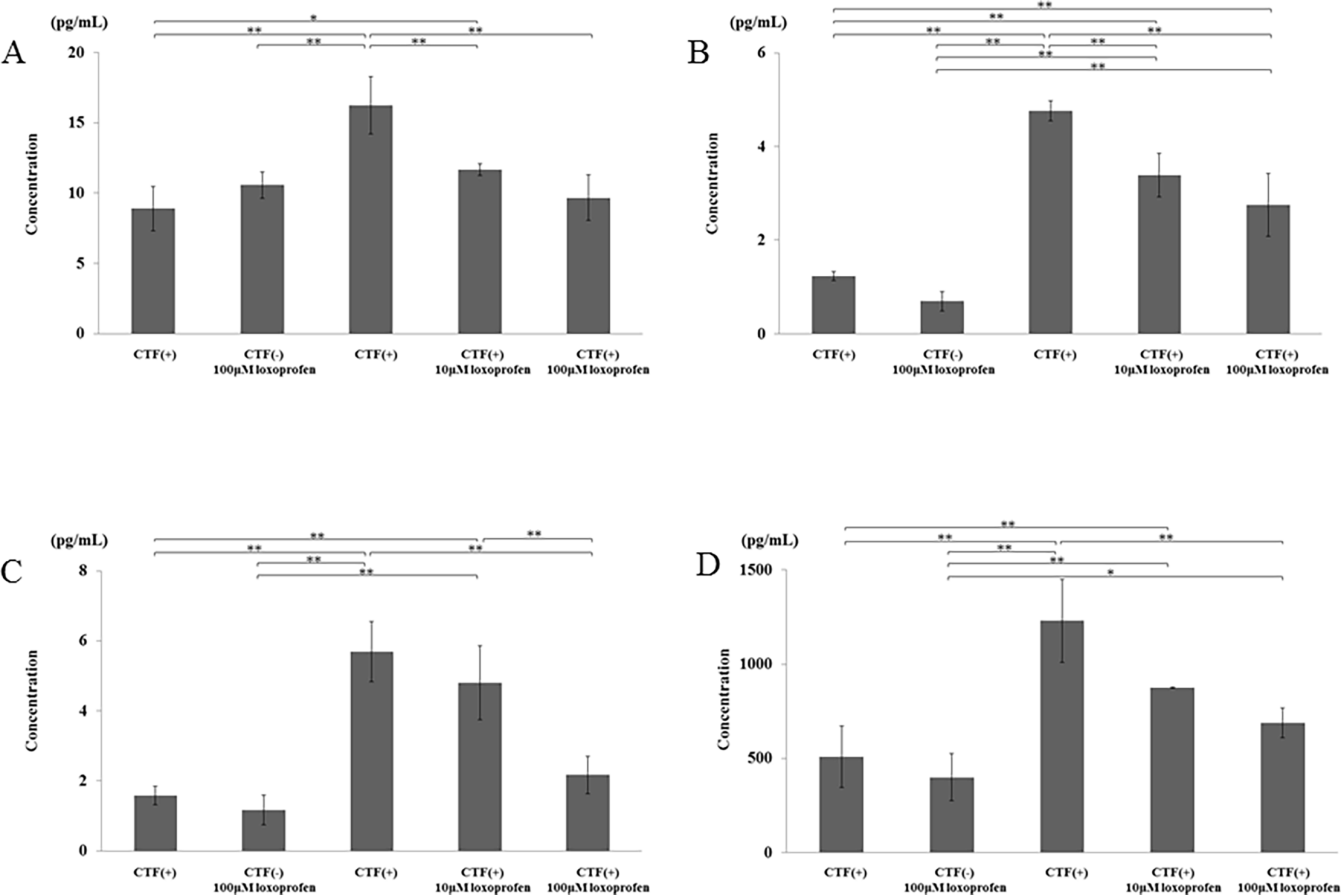
The effects of loxoprofen sodium on the expression of IL-1β, TNF-α, RANKL and M-CSF protein concentrations in pulp cells stimulated by a 10-kPa CTF for 48 hours. (A) Significant decreases in IL-1βconcentration were observed in the 10- and 100-μM loxoprofen groups compared with the CTF group. (B) Protein concentration of TNF-α, (C) RANKL and (D) M-CSF was decreased significantly in the 100-μM loxoprofen group compared with the CTF group (**P< 0.01, N = 4).

The protein concentration of IL-1β, TNF-α, RANKL, and M-CSF was increased by the application of 10-kPa CTF in comparison to the control group. Protein concentrations of all factors were decreased by loxoprofen administration in a dose-dependent manner. The amount of IL-1β concentration was significantly reduced by 10- and 100-μM loxoprofen (p < 0.01, Fig 6A). The protein concentration of TNF-α, RANKL, and M-CSF was also decreased significantly by 100-μM loxoprofen (p < 0.01, Fig 6B, 6C and 6D). There was no significant difference between the control group and CTF(-) with 100-μM loxoprofen group in the protein expression level of all these factors (Fig 6A–6D).

### The amount of tooth movement

**Fig 7:**
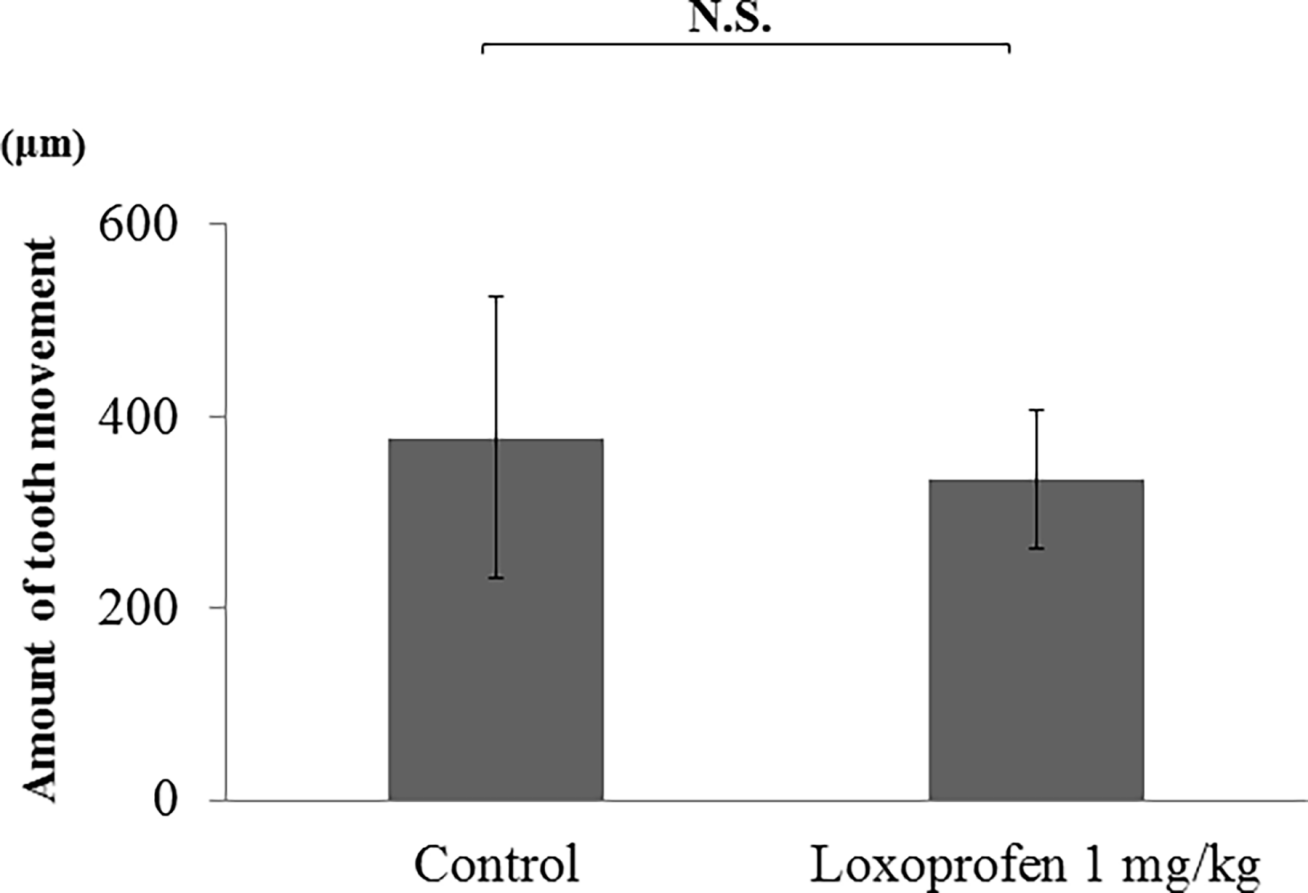
Changes in the amount of tooth movement. There was no significant difference in the amount of tooth movement between the control and loxoprofen groups.

After 30 days, the amount of tooth movement was measured through computed tomography, and the amount of apical root resorption was determined. No significant difference were observed in the amount of tooth movement between the control and loxoprofen groups (Fig 7).

### Immunohistochemical staining and histological examination, and the amount of root resorption

**Fig 8:**
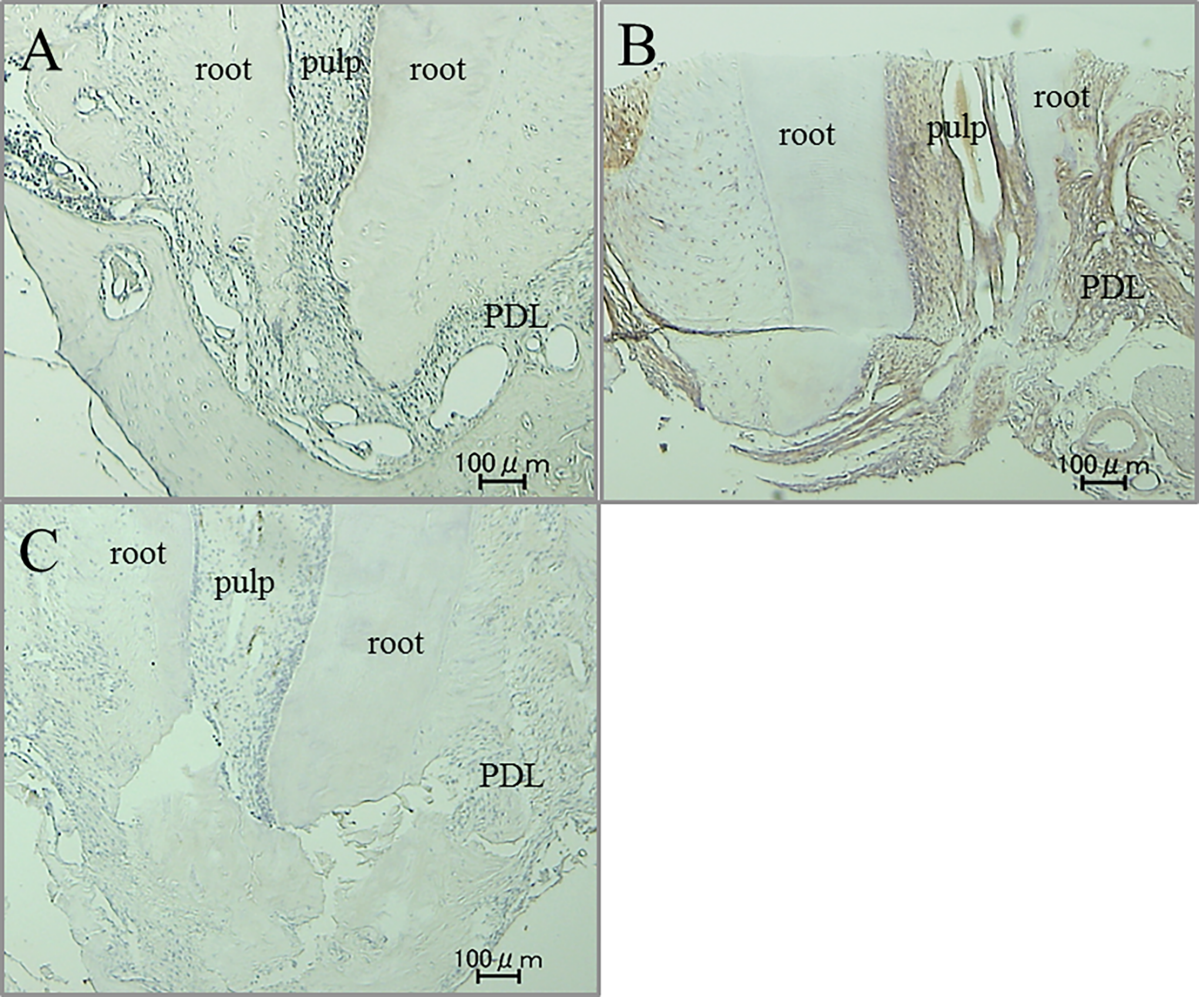
Immunohistochemical staining for IL-1β. (A) non-tooth movement without drug (B) tooth movement without drug (C) tooth movement with loxoprofen.

**Fig 9:**
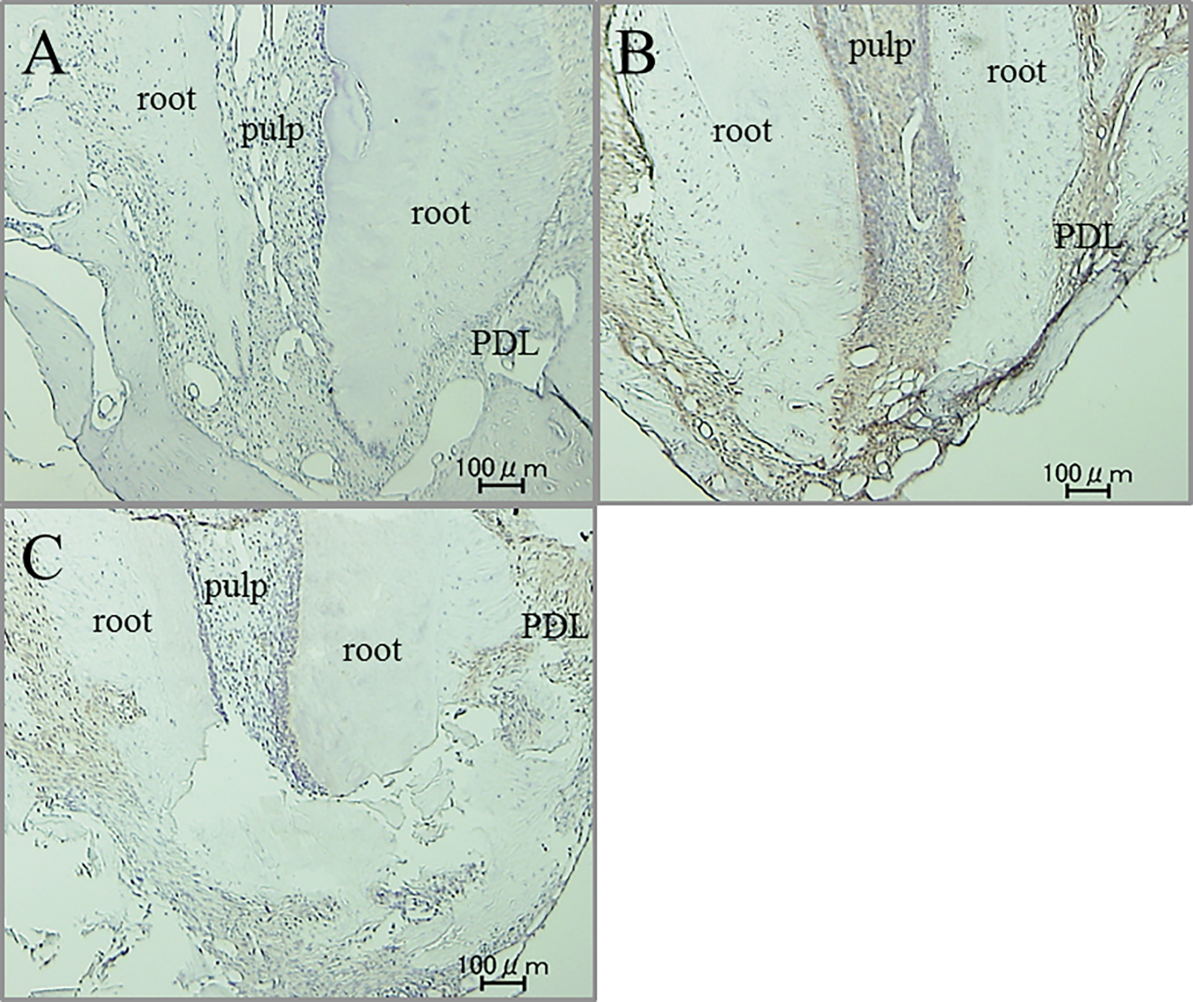
Immunohistochemical staining for TNF-α. (A) non-tooth movement without drug (B) tooth movement without drug (C) tooth movement with loxoprofen.

**Fig 10:**
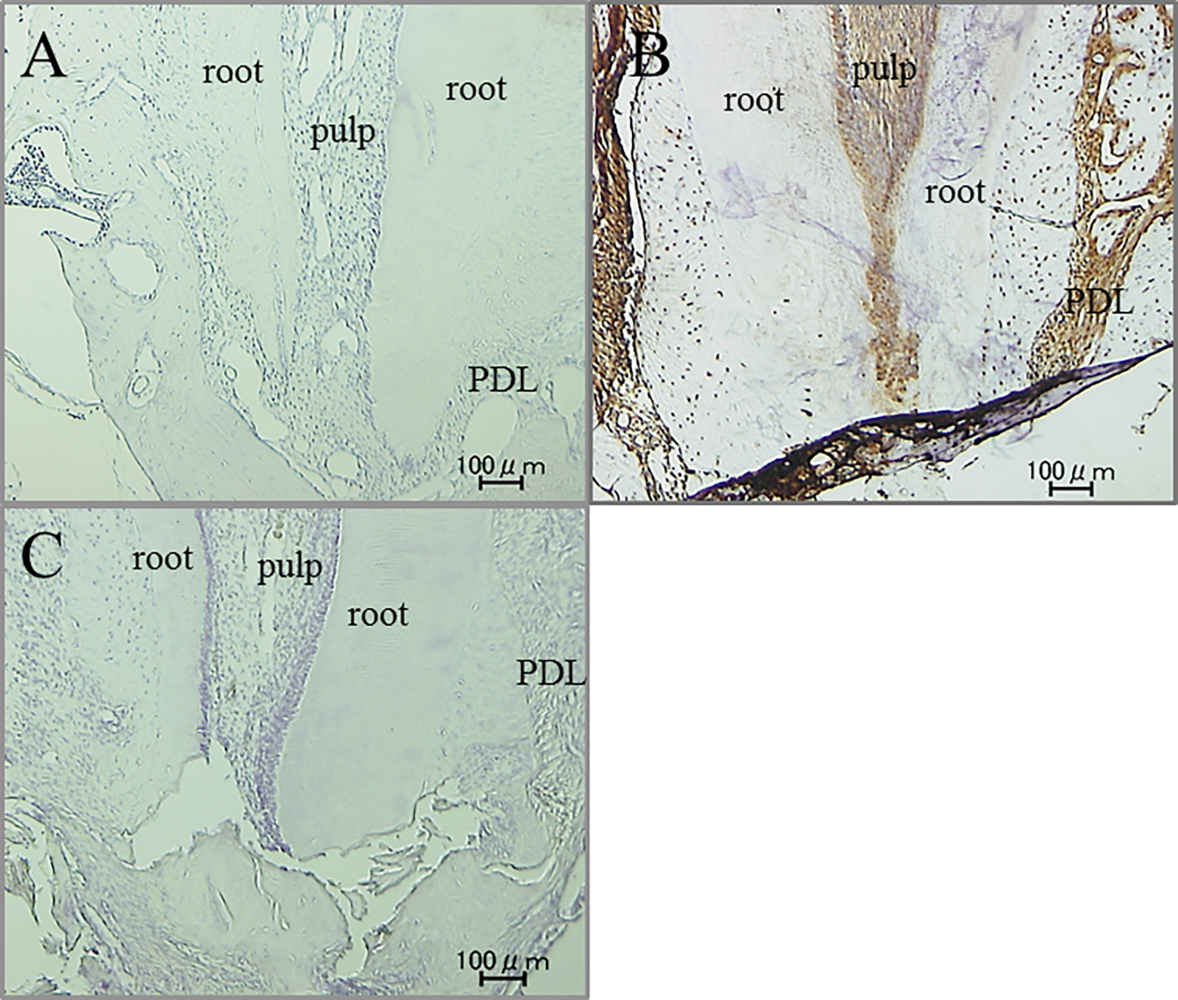
Immunohistochemical staining for RANKL. (A) non-tooth movement without drug (B) tooth movement without drug (C) tooth movement with loxoprofen.

**Fig 11:**
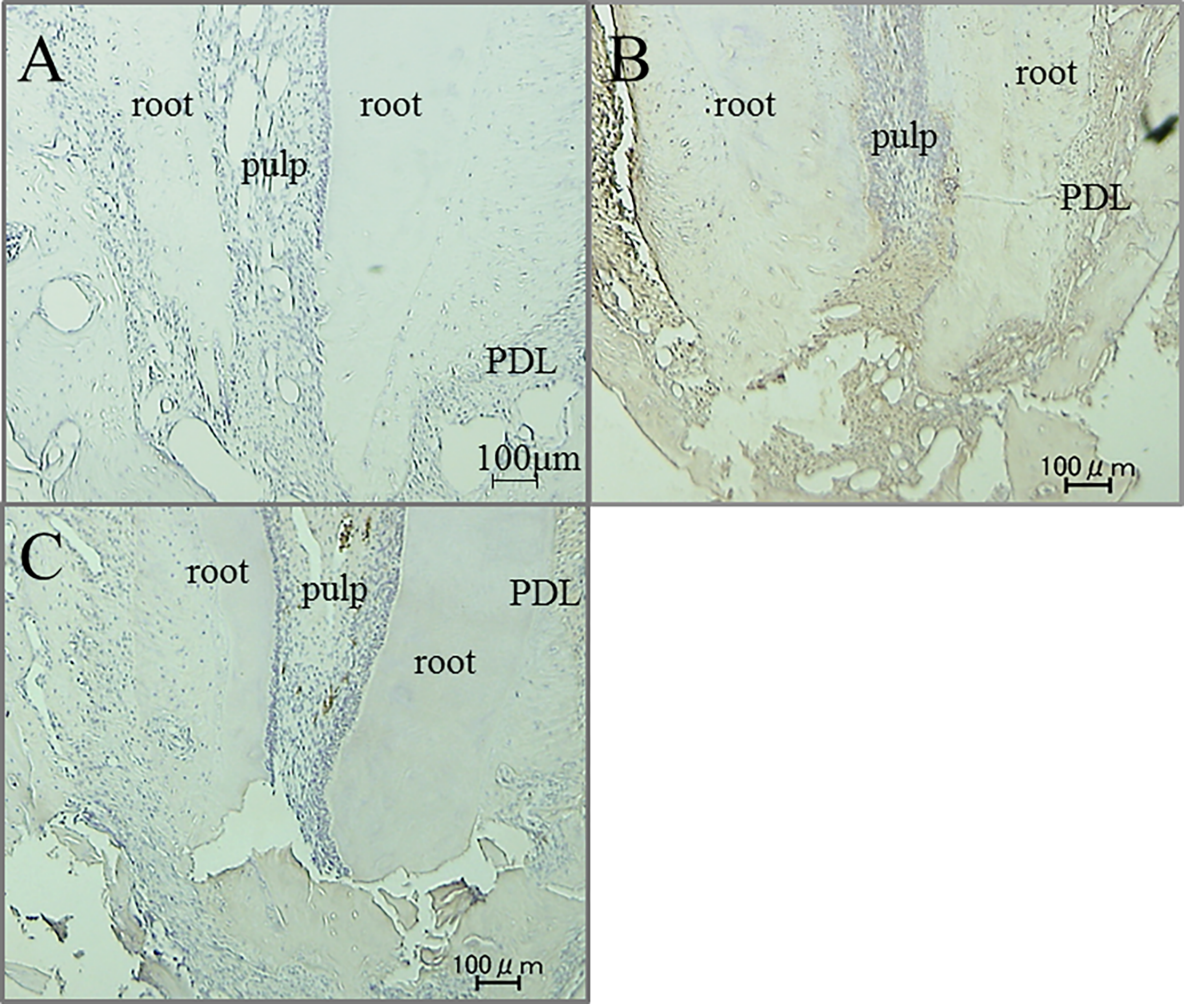
Immunohistochemical staining for M-CSF. (A) non-tooth movement without drug (B) tooth movement without drug (C) tooth movement with loxoprofen.

Immunohistochemical activity of IL-1β, TNF-α, RANKL and M-CSF was investigated 30 days after tooth movement. In the non-tooth movement without pharmacological treatment group, positive cells of these factors were hardly seen in apical pulp and periodontal ligament (PDL) tissue (Figs 8A, 9A, 10A and 11A). Immunoreactivity of these factors were detected in apical pulp and PDL tissue after tooth movement (Figs 8B, 9B, 10B and 11B), meanwhile, the expression of these factors in the loxoprofen groups decreased in apical pulp and PDL tissue, but TNF-α and M-CSF in PDL tissue (Figs 8C, 9C, 10C and 11C).

**Fig 12:**
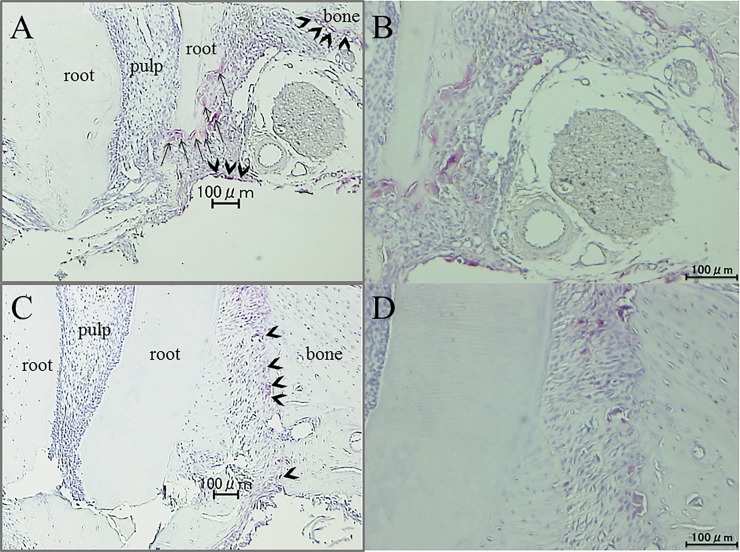
Histologic examination after orthodontic tooth movement. (A) control group (tooth movement without drug, arrows indicate odontoclasts in the apical root area and arrow heads indicate osteoclasts in the alveolar bone), (B) magnification of control group (C) loxoprofen group (tooth movement with loxoprofen, arrow heads indicate osteoclasts in the alveolar bone). (D) magnification of loxoprofen group, Osteoclasts were found in the pressure side of alveolar bone after tooth movement both in the control and loxoprofen groups. Many odontoclasts appeared at the apical root area in the control group, although there was no odontoclast in the loxoprofen group.

**Fig 13:**
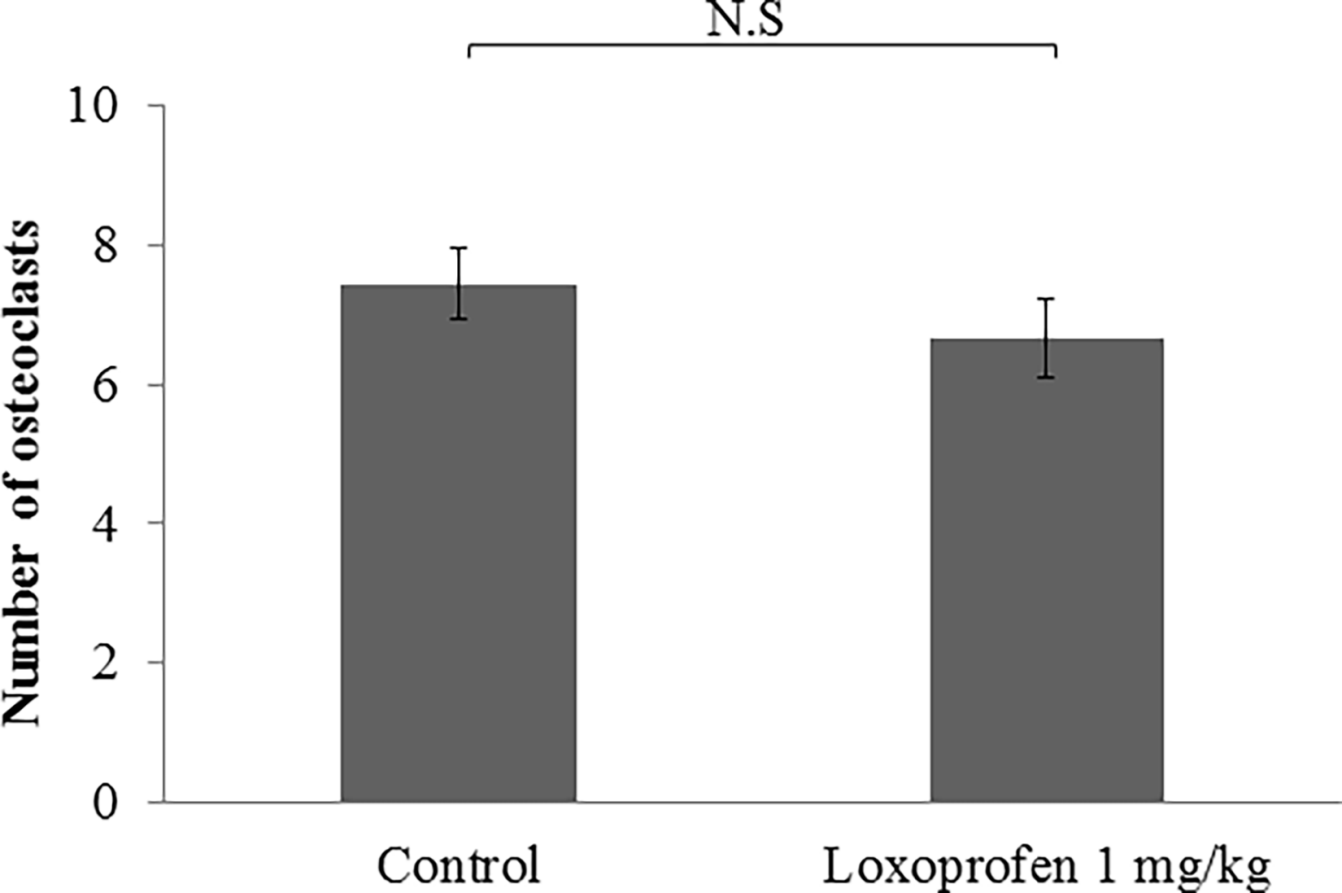
The number of osteoclasts after orthodontic tooth movement. No statistical different was observed between control group (tooth movement without drug) and loxoprofen group (tooth movement with loxoprofen).

**Fig 14:**
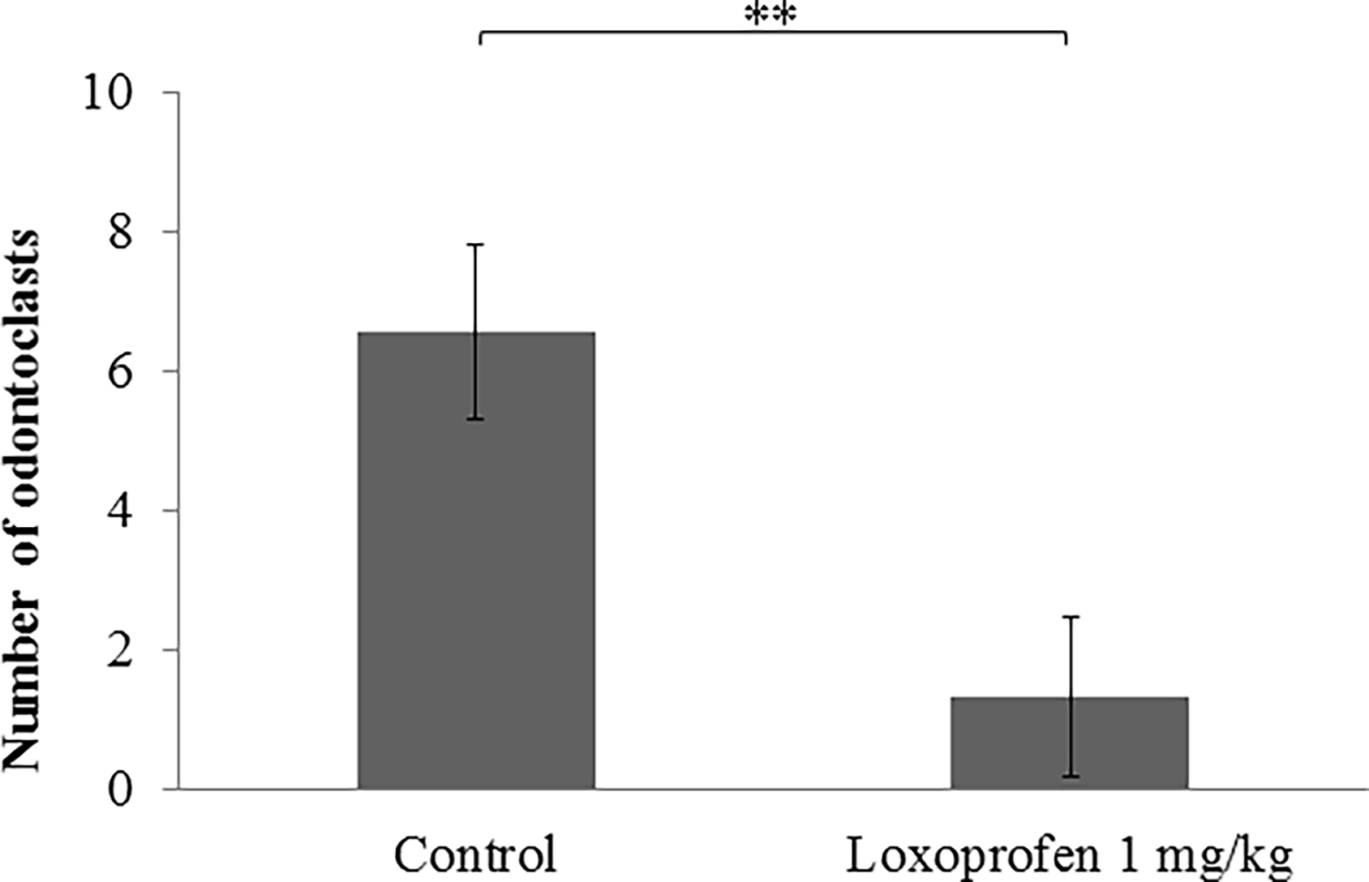
The number of odontoclasts at the root apex after orthodontic tooth movement. The number of odontoclasts in the control group (tooth movement without drug) was significantly heigher than in the loxoprofen group (tooth movement with loxoprofen) (**P< 0.01, N = 5).

**Fig 15:**
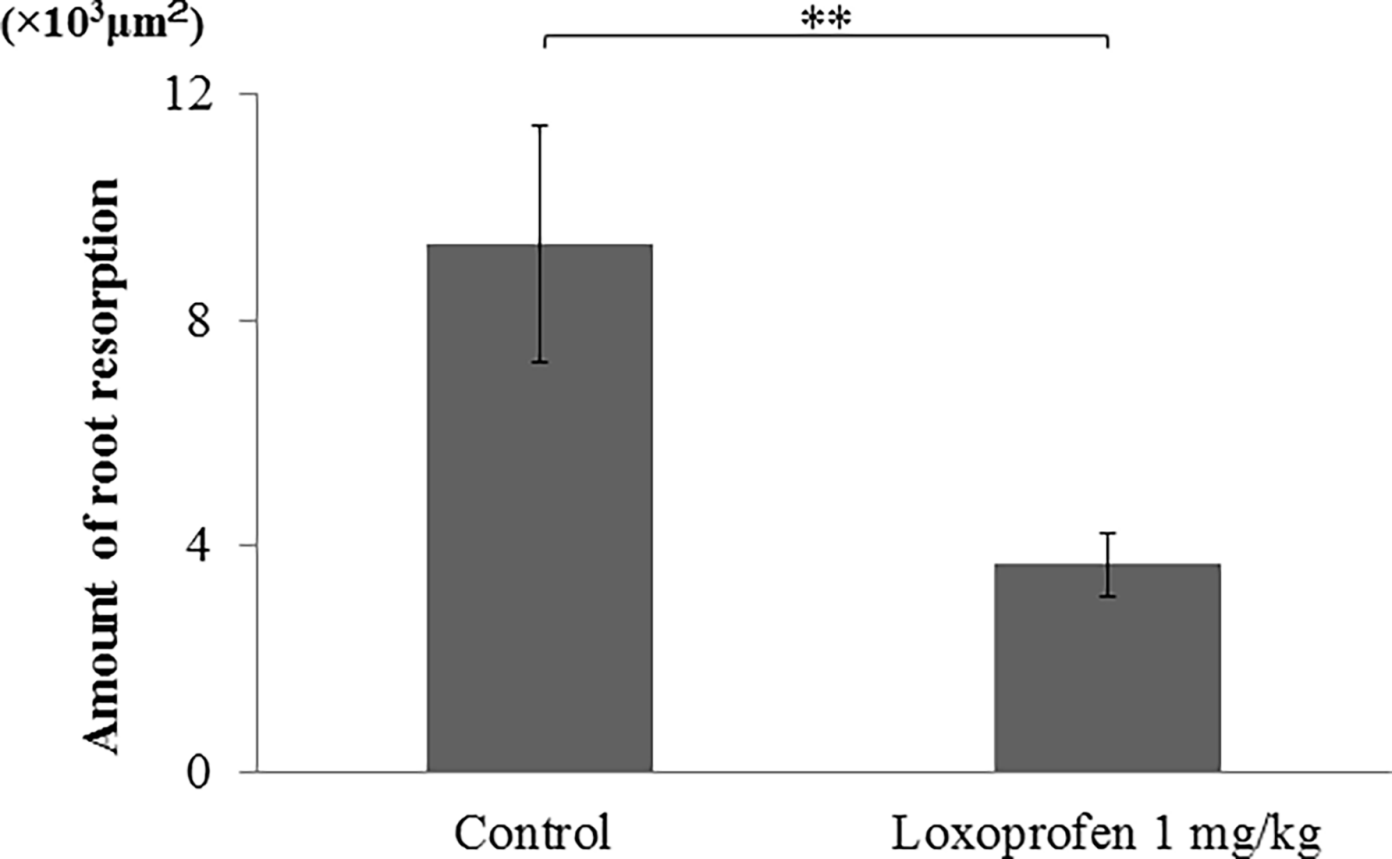
The amount of root resorption. The amount of root resorption was significantly greater in the control group (tooth movement without drug) than in the loxoprofen group (tooth movement with loxoprofen) (**P< 0.01, N = 5).

Many osteoclasts were seen in the pressure side of alveolar bone after tooth movement both with or without loxoprofen group (Fig 12A–12D). There is not statistically different in the number of osteoclasts in the pressure side of alveolar bone between the two groups (Fig 13). Many odontoclasts and reabsorption lacunae were observed at the root apices in the control group. In the loxoprofen group, odontoclasts and reabsorption lacunae were hardly observed at the apical root areas (Fig 12A–12D). The number of odontoclasts was significantly less in the loxoprofen group than in the control group (Fig 14). The amount of root resorption at the root apex was significantly less in the loxoprofen group compared with the control group (Fig 15).

## Discussion

Many previous studies have examined the relationship between orthodontic tooth movement and root resorption, and numerous causes are suggested as risk factors for root resorption. As host factors, systemic conditions, patient age [2], root shape, and alveolar bone density [17] were reported to be candidates. For treatment factors, it is thought that the amount of root resorption highly depends on force mechanics and treatment period [2]. In contrast, the amount of root resorption has been reported to be less in pulpectomized teeth than in vital teeth [8, 9]. Excessive orthodontic force may impair the apical vessels and stretching them by teeth tipping may cause pulpal disturbance [18]. McDonald *et al*. suggested that continuous tipping force application reduced pulpal blood vessels [7], and it was also demonstrated that continuous heavy orthodontic forces lead to pulp necrosis by the blood vessel rupturing at the root apex [6]. Furthermore, Bender *et al*. reported that periapical resorption after orthodontic treatment is a frequent and unpredictable symptom, and they indicated that vital teeth showed severe apical root resorption than did endodontically treated teeth, although the role of the vital pulp in apical root resorption was unclear [19]. Recently, we showed that the gene and protein expression of IL-1β, TNF-α, RANKL, and M-CSF in human pulp cells are increased by the application of 10-kPa CTF after 48 hours [10]. We also demonstrated that in the experimental rat model of tooth movement, the expression of these factors was upregulated in vital teeth compared with pulpectomized teeth [11], and the number of odontoclasts and amount of root resorption around the root apex was significantly high in vital teeth than in pulpectomized teeth [10, 11]. These findings strongly suggested that severe apical root resorption occurs due to noninfective inflammatory reaction at apical root pulp tissue during orthodontic tooth movement.

IL-1β and TNF-α are believed to have a strong effect on osteoclast differentiation. It was reported that IL-1β can stimulate osteoclast differentiation and bone-resorbing activity [20], and TNF-α supports the fusion of monocytes into osteoclasts [21]. Kodama *et al*. [22] observed that osteopetrosis in osteopetrotic (*op/op*) mice is curable with an increase in osteoclasts by administration of recombinant human M-CSF. Therefore, it was clear that M-CSF plays a crucial role in osteoclast differentiation. RANKL is also a crucial factor for osteoclast differentiation and activation [23], and M-CSF can enhance osteoclast differentiation in cooperation with RANKL [24]. In this study, the gene and protein expression of IL-1β, TNF-α, RANKL, and M-CSF was increased by 10-kPa CTF in the loxoprofen group in comparison to the control group. Thus, it was proved that the stretched and injured pulp cells enhance the expression of these factors.

NSAIDs are the most commonly used drugs for pain, inflammation, and fever [25], and the therapeutic effects are produced by the inhibition of COX activity [26]. Loxoprofen is a nonselective COX inhibitor, which is widely prescribed in the clinic, and it provides effective pain relief for inflammatory conditions. Because loxoprofen is a prodrug, it does not show a prostaglandin-inhibitory effect before being absorbed in the body through the stomach. Therefore, loxoprofen can exhibit an anti-inflammatory reaction with little adverse effects, such as gastrointestinal dysfunction [27]. In this study, the gene expression of COX-1, COX-2, IL-1β, TNF-α, RANKL, and M-CSF, which were upregulated by CTF, were significantly decreased in a dose-dependent manner by loxoprofen administration. Furthermore, De Boer *et al*. reported that *ex vivo* release of IL-1β and TNF-α levels in synovial tissue from patients with knee osteoarthritis were decreased by COX inhibitor treatment [28]. So, our results suggested that loxoprofen can decrease the expression of CTF-induced IL-1β and TNF-α by blocking COX activity in pulp cells.

Several investigators have demonstrated that root resorption worsens with an increase in the force magnitude [29, 30]. Gonzales *et al.* [31] reported that the amount of tooth movement was significantly larger with notable less root absorption in a light force application group (10*g* force application for rat molar) compared with that in a heavy force application group of rats during an experimental period of 28 days. Kohno *et al*. [32] showed that the optimal orthodontic force for rat upper molars was less than 10*g*. Therefore, in this study, we applied a 10*g* force for the molar mesial movement to determine the number of odontoclasts and amount of root resorption. Jerome *et al*. [33] showed that the selective COX-2 inhibitor celecoxib did not interfere with tooth movement, and they found a significantly lower number of root lacunaes and amount of root resorption after celecoxib administration in rats. Our results also revealed many osoteoclasts on the pressure side of the alveolar bone in the loxoprofen group, and there was no significant difference in the amount of tooth movement between the control and loxoprofen groups. The number of odontoclasts was significantly less in the loxoprofen group compared with the control group, and the amount of root resorption at the root apex was significantly less in the loxoprofen group in comparison to the control group. The following points can explain why loxoprofen does not disturb tooth movement and prevent root resorption. One of the causes is the different compositions of the root and alveolar bone. Because dentin and cementum are mainly matrix-dominant tissues without blood vessels in contrast to alveolar bone [34, 35], it is thought that there are few supplies of odontclast precursor from peripheral blood vessel. Another reason is a protective effect of dental hard tissue conferred by cementoblast. Kurihara reported that because the cementum is protected by cementoblasts, it is believed that the absorption of its root is arduous during orthodontic tooth movement. When excessive mechanical stress is applied, atrophy and migration of cementoblasts on the cementum occur and then, increased chemotaxis of odontoclasts into the exposed cementum can result in root resorption. On the other hand, bone tissue is much easy to be resorbed during orthodontic tooth movement by migration of osteoclasts [36]. Furthermore, our results in this study exhibited that TNF-α and M-CSF in PDL tissue of pressure side expressed even in the loxoprofen groups, however, the expression of IL-1β, TNF-α, RANKL, and M-CSF in apical pulp tissue decreased in the loxoprofen groups. From these findings, it is supposed that apical root resorption with inflammatory reaction can be suppressed by loxoprofen administration, although the dosage is not sufficient to avoid migration of osteoclasts in alveolar bone of the pressure side. However, further clinical investigation will be needed to clarify the effect of NSAIDs on the amount of tooth movement and apical root resorption.

## Conclusions

Gene expression and protein concentration of IL-1β, TNF-α, RANKL, and M-CSF were significantly higher in the CTF group than those in the control group and these levels decreased by loxoprofen administration. Less number of odontoclasts and decrease in the amount of apical root resorption were observed in loxoprofen group after orthodontic tooth movement. However, there was no significant difference in the amount of tooth movement between the control and loxoprofen group. These findings demonstrate that severe apical root resorption may be suppressed by loxoprofen administration without disturbance of tooth movement.
